# Video Traffic Characteristics of Modern Encoding Standards: H.264/AVC with SVC and MVC Extensions and H.265/HEVC

**DOI:** 10.1155/2014/189481

**Published:** 2014-02-20

**Authors:** Patrick Seeling, Martin Reisslein

**Affiliations:** ^1^Department of Computer Science, Central Michigan University, Mount Pleasant, MI 48859, USA; ^2^School of Electrical, Computer, and Energy Engineering, Arizona State University, Tempe, AZ 85287-5706, USA

## Abstract

Video encoding for multimedia services over communication networks has significantly advanced in recent years with the development of the highly efficient and flexible H.264/AVC video coding standard and its SVC extension. The emerging H.265/HEVC video coding standard as well as 3D video coding further advance video coding for multimedia communications. This paper first gives an overview of these new video coding standards and then examines their implications for multimedia communications by studying the traffic characteristics of long videos encoded with the new coding standards. We review video coding advances from MPEG-2 and MPEG-4 Part 2 to H.264/AVC and its SVC and MVC extensions as well as H.265/HEVC. For single-layer (nonscalable) video, we compare H.265/HEVC and H.264/AVC in terms of video traffic and statistical multiplexing characteristics. Our study is the first to examine the H.265/HEVC traffic variability for long videos. We also illustrate the video traffic characteristics and statistical multiplexing of scalable video encoded with the SVC extension of H.264/AVC as well as 3D video encoded with the MVC extension of H.264/AVC.

## 1. Introduction

Network traffic forecasts, such as the Cisco Visual Networking Index [1], predict strong growth rates for video traffic. Typical predicted annual growth rates are 30% or higher for wireline IP-based video services and 90% for Internet TV in mobile networks. Due to these high growth rates, video traffic will account for a large portion of the traffic in communication networks. Specifically, the estimates by Cisco, Inc. predict that video will contribute close to two-thirds of the mobile network traffic by 2014. Network designers and engineers therefore need a basic understanding of video traffic in order to account for the video traffic characteristics in designing and evaluating communication services for this important type of network traffic.

The encoders that are used to compress video before network transport have significantly advanced in recent years. These video encoding advances have important implications for the network transport of encoded video. The purpose of this paper is to first give an overview of the recent developments in video coding. Then, we examine the traffic characteristics of long videos encoded with the recently developed video coding standards so as to illustrate their main implications for the transport of encoded video in communication networks.

This paper covers three main areas of video coding advances: (i) efficient encoding of conventional two-dimensional (2D) video into a nonscalable video bitstream, that is, a video bitstream that is not explicitly designed to be scaled (e.g., reduced in bitrate) during network transport, (ii) scalable video coding, that is, video coding that is explicitly designed to permit for scaling (e.g., bitrate reduction) so as to make the video traffic adaptive (elastic) during network transport, and (iii) efficient nonscalable encoding of three-dimensional (3D) video. For conventional 2D video, we start from the video coding standards MPEG-2 and MPEG-4 Part 2 and outline the advances in video coding that have led to the H.264/MPEG-4 Advanced Video Coding (H.264/AVC) standard (formally known as ITU-T H.264 or ISO/IEC 14496-10) [2] as well as the High Efficiency Video Coding (H.265/HEVC) standard [3–5]. We also briefly review the Scalable Video Coding (SVC) extension [6] of H.264/AVC, which is commonly abbreviated to H.264 SVC. For 3D video, we consider the Multiview Video Coding (MVC) standard [7, 8], formally Stereo and Multiview Video Coding extension of the H.264/MPEG-4 AVC standard.

Video-coding-specific characteristics and performance metrics of these latest video coding standards are covered for nonscalable coding (H.264/AVC and H.265/HEVC) in [5, 9–13] and for scalable video coding (H.264/AVC with SVC extension) in [6, 14]. The evaluations in this existing literature focus primarily on the rate-distortion (RD) characteristics of the video encoding, that is, the video quality (distortion) as a function of the mean bitrate of an encoded video stream, for relatively short video sequences (typically up to 10 s). Complementary to these existing evaluations, this paper considers long video sequences (of 10 minutes or more) and includes evaluations of the variability of the encoded video traffic as well as network link bitrates for statistical multiplexing, which are key concerns for network transport. Previous studies have considered long videos only for video coding standards preceding H.265/HEVC. For instance, the traffic of long H.264/AVC and H.264 SVC encoded videos has been studied in [15–17]. To the best of our knowledge, the traffic characteristics of H.265/HEVC for long videos are for the first time examined in this present study.

A basic understanding of video coding standards and the resulting video traffic characteristics is required for a wide range of research topics in communications and networking. Communications and networking protocols, for instance, need to conform with the timing constraints arising from the frame dependencies introduced by the video encoding and accommodate the video traffic with its bitrate variability. Wireless mobile networks [18–26], sensor networks [27–31], peer-to-peer networks [32–35], and metro/access networks [36–42] will likely experience large volumes of encoded video traffic. Also, streaming of 3D video has begun to attract research attention (see, e.g., [43–46]) and will likely contribute large volumes of traffic in future communication networks.

**Figure 1:**
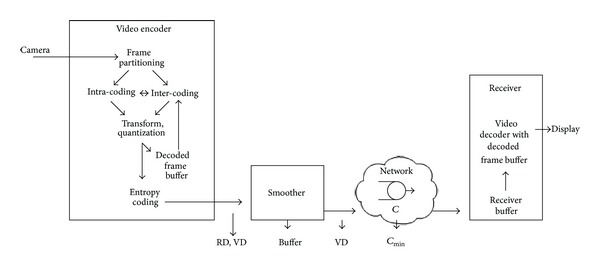
Block diagram of video network transport system. The captured video frames are encoded and smoothed before network transmission. The evaluations of video transmission consider the rate-distortion (RD) characteristics, the rate variability-distortion (VD) characteristics before and after the smoother, and the required smoother buffer and the link bitrate *C*_min⁡_ requirements.

The structure of this paper follows the block diagram of a video network transport system in Figure 1. The main acronyms and notations in this paper are summarized in Table 1. The video is captured by a camera and encoded (compressed) using encoding mechanisms. The advances in video coding mechanisms that have led to H.265/HEVC are reviewed in Section 2. In Section 3, we examine the RD and rate variability-distortion (VD) characteristics of the encoded frames as they appear at the output of the video encoder. We then give an overview of the network transport of encoded video. The encoded video bitstream is typically very bursty; that is, the video traffic bitrate exhibits high variability. The traffic is therefore commonly passed through a smoother before network transmission, as illustrated in Figure 1. We characterize the required smoother buffer and compare the VD characteristics after smoothing with the VD characteristics at the encoder output (i.e., before the smoother). We also examine the minimum required link bitrate *C*_min⁡_ for the statistical multiplexing of a fixed number of video streams. Details of the receiver processing and video display are beyond the scope of this paper. In Sections 4 and 5, we give overviews of the encoding and network transport of scalable encoded video. In Section 6, we examine 3D video encoding and transport.

Video traces for the areas of video encoding covered in this paper are available from http://trace.eas.asu.edu/ [47]. Video traces characterize the encoded video through plain text files that provide the sizes of encoded frames and the corresponding video quality (distortion) values. The video traces facilitate traffic modeling, as well as the evaluation of a wide range of video transport paradigms.

## 2. Overview of Nonscalable Video Encoding

We first give a brief overview of the main encoding steps in the major video coding standards and then review the advances in these main encoding steps. In the major video coding standards, a given video frame (picture) is divided into blocks. The blocks are then intracoded, that is, encoded by considering only the current frame, or intercoded, that is, encoded with references to (predictions from) neighboring frames that precede or succeed the current frame in the temporal display sequence of the frames. The inter-coding employs motion-compensated prediction, whereby blocks in the reference frames that closely resemble the considered block to be encoded are found; the considered block is then represented by motion vectors to the reference blocks and the prediction errors (differences between the considered block and the reference blocks). The luminance (brightness) and chrominance (color) values in a block, or the corresponding prediction errors from reference blocks, are transformed to obtain a block of transform coefficients. The transform coefficients are then quantized, whereby the quantization is controlled by a quantization parameter (QP), and the quantized values are entropy coded. The entire sequence of encoding steps is commonly optimized through RD optimization, which has advanced along with the individual encoding steps.

**Figure 2:**
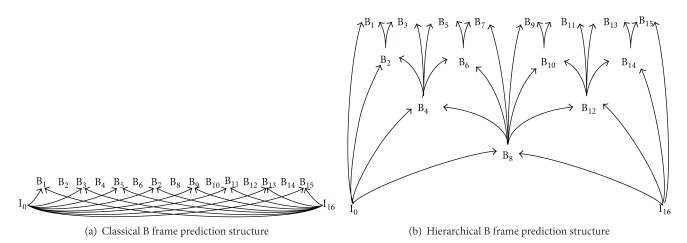
Illustration of classical B frame prediction structure used in MPEG-2 and MPEG-4 Part 2 (without reference arrows for even frames to avoid clutter) and dyadic hierarchical B frame prediction structure of H.264/AVC, H.264 SVC, and H.265/HEVC.

MPEG-2, which is formally referred to as ISO/IEC standard 13818-2 and ITU-T recommendation H.262 and is also known as MPEG-2 Video or MPEG-2 Part 2, introduced three frame types that are also used in the subsequent coding standards: intracoded (I) frames are encoded as stand-alone pictures without references (dependencies) to other frames. Predictive-coded (P) frames are encoded with inter-coding with respect to only preceding I (or P) frames in the temporal frame display order. Bi-directional-coded (B) frames are intercoded with respect to both preceding (i.e., past) and succeeding (i.e., future) I (or P) frames, as illustrated in Figure 2(a). A group of frames (pictures) from one I frame to the frame immediately preceding the next I frame is commonly referred to as a group of pictures (GoP).  Figure 2 considers a GoP structure with 15 B frames between successive I frames (and without any P frames).

### 2.1. Frame Partitioning into Blocks and Intra-Coding of Video Frames

As the video coding standards advanced, the partitioning of a video frame (picture) into blocks has become increasingly flexible to facilitate high RD efficiency in the subsequent coding steps. While MPEG-2 was limited to a fixed block size of 16 × 16 luminance pixels, MPEG-4 (formally ISO/IEC 14496-2, also known as MPEG-4 Part 2 or MPEG-4 Visual) permitted 16 × 16 and 8 × 8 blocks, and H.264/AVC introduced block sizes ranging from 4 × 4 to 16 × 16. High Efficiency Video Coding (H.265/HEVC, formally H.265/MPEG-H Part 2) [5] introduces frame partitioning into coding tree blocks of sizes 16 × 16, 32 × 32, and 64 × 64 luminance pixels which can be flexibly partitioned into multiple variable-sized coding blocks.

While preceding standards had few capabilities for intra-coding within a given frame, H.264/AVC introduced spatial intra-coding to predict a block in a frame from a neighboring block in the same frame. H.265/HEVC significantly advances intra-coding through the combination of the highly flexible coding tree partitioning and a wide range of intra-frame prediction modes.

### 2.2. Inter-Coding (Temporal Prediction) of Video Frames

Advances in inter-coding, that is, the encoding of frames with motion-compensated prediction from other frames in the temporal frame display sequence, have led to highly significant RD efficiency increases in the advancing video coding standards. In MPEG-2 and MPEG-4 Part 2, B frames are predicted from the preceding I (or P) frame and the succeeding P (or I) frame; see Figure 2(a). Compared to the motion-compensated prediction at half-pixel granularity in MPEG-2, MPEG-4 Part 2 employs quarter-pixel granularity for the motion-compensated prediction as well as additional RD efficiency increasing enhanced motion vector options and encoding. H.264/AVC and H.265/HEVC employ similarly quarter-pixel granularity for the motion-compensated prediction and further improve the motion parameters.

H.264/AVC and H.265/HEVC fundamentally advance inter-coding by predicting B frames from potentially multiple past and/or future B frames. Specifically, in H.264/AVC (and H.264 SVC) as well as H.265/HEVC, the frames in a GoP are capable of forming a dyadic prediction hierarchy illustrated in Figure 2(b). I frames (and P frames, if present in the GoP) form the base layer of the hierarchy. With *β* B frames between successive I (or P) frames, whereby *β* = 2^*τ*^ − 1 for a positive integer *τ* for the dyadic hierarchy, the B frames form *τ* = log⁡_2_(*β* + 1) layers. For instance, in the GoP structure with 15 B frames (and no P frames) between successive I frames, illustrated in Figure 2(b), the *β* = 15 B frames in the GoP structure form *τ* = 4 layers. A B frame in a layer *n*, 1 ≤ *n* ≤ *τ*, is intercoded with reference to the immediately preceding and succeeding frames in lower layers *n* − 1, *n* − 2,…, 0, whereby layer 0 corresponds to the base layer. For instance, frame B_3_ is encoded through motion-compensated prediction with reference to frames B_2_ and B_4_, while frame B_2_ is encoded with reference to frames I_0_ and B_4_. A wide variety of alternative GoP structures can be produced to accommodate different application scenarios.

### 2.3. Quantization, Transform, and Entropy Coding

MPEG-2 and MPEG-4 Part 2 allow for different quantization parameter (QP) settings for the three different frame types, namely, I, P, and B frames. Generally, it is an RD-efficient coding strategy to quantize I frames relatively finely, that is, with a small QP, since the I frames serve as a reference for the P and B frames. Increasingly coarse quantization, that is, successively larger QPs, for P and B frames can increase RD efficiency, since P frames serve only as reference for B frames and B frames are not employed as reference for inter-coding in MPEG-2 and MPEG-4 Part 2; that is, no other frames depend on B frames. In H.264/AVC and H.265/HEVC, this principle of increasingly coarse quantization for frames with fewer dependent frames can be pushed further by increasing the QP with each level of the frame hierarchy. This strategy is commonly referred to as QP cascading and is examined quantitatively for H.264/AVC in Section 3.

MPEG-2 and MPEG-4 Part 2 employ the discrete cosine transform (DCT) on blocks of 8 × 8 samples. H.264/AVC provides more flexibility with 4 × 4 and 8 × 8 transforms and H.265/HEVC further significantly increases the flexibility with transforms that match the flexibility of the code tree block structure (i.e., 4 × 4 up to 32 × 32).

MPEG-2 and MPEG-4 Part 2 employ a basic variable-length coding of the coefficients resulting from the DCT (after quantization). H.264/AVC introduced more efficient context-adaptive variable-length coding (CAVLC) and context-adaptive binary arithmetic coding (CABAC), whereby CABAC achieves typically higher RD efficiency than CAVLC [48]. H.265/HEVC employs CABAC with refined context selection.

### 2.4. Error Resilience

Many communication networks provide unreliable best-effort service; that is, packets carrying parts of the encoded video bit stream data may be lost or corrupted during network transport. Depending on the networking scenario, the communication network may employ channel coding to protect the video bitstream from errors or may employ loss recovery mechanisms, such as retransmissions to recover lost or corrupted packets. At the same time, the advancing video coding standards have incorporated provisions for forward error control mechanisms in the video encoder and complementary error concealment mechanisms in the video decoder, which we now briefly review. For more details we refer to [2, 49, 50].

One key error control and concealment mechanism is slices that encode different regions (usually horizontal slices) of a given video frame (picture). The slices encoding a picture have essentially no encoding dependencies and can be transmitted in separate packets. Thus, loss of a packet carrying a slice still permits decoding of the other slices of the picture. The slice concept originated in the earlier MPEG codecs and was retained in H.264/AVC and H.265/HEVC.

H.264/AVC introduced a wide range of error control and concealment mechanisms, such as redundant slices, arbitrary slice order (ASO), and flexible macroblock order (FMO), as well as special SP/SI frame types [2]. These mechanisms have rarely been used in practice and have therefore not been included in H.265/HEVC. On the other hand, H.265/HEVC adopted and expanded some key error concealment mechanisms of H.264/AVC. For instance, H.264/AVC introduced supplemental encoding information (SEI) messages that aid the decoder in detecting scene changes or cuts. Accordingly, the decoder can then employ suitable error concealment strategies. H.265/HEVC introduces a new SEI message for a checksum of the decoded picture samples, which aids in error detection. Moreover, a new SEI message gives a structure of pictures (SOP) description that indicates the interprediction and temporal structure of the encoding. The decoder can use the SOP information to select the error concealment strategy appropriate for the extent of temporal loss propagation.

H.265/HEVC has a new reference picture set (RPS) concept for the management of reference pictures. Whereas preceding standards signaled only relative changes to the set of reference pictures (making it vulnerable to missing a change due to lost/corrupted packets), H.265/HEVC signals the (absolute) status of the set of reference pictures. Similarly, H.265/HEVC improved error resilience through a new video parameter set (VPS) concept for signaling essential syntax information for the decoding.

Generally, stronger compression achieved by more advanced encoding mechanisms makes the encoded video bit stream more vulnerable to packet corruption and losses than less sophisticated compression with lower RD efficiency. The H.265/HEVC error control and concealment mechanisms can provide a basic level of error resilience. The detailed evaluation of H.265/HEVC error resilience mechanisms, including their impact on the RD coding efficiency and their use in conjunction with channel coding and network loss recovery mechanisms, is an important direction for future research and development.

## 3. Network Transport of Nonscalable Encoded Video

### 3.1. Video Network Transport Scenarios

Main scenarios for the transport of encoded video over networks are download or streaming of prerecorded content and live video transmission. For download, the entire prerecorded and encoded video is received and stored in the receiver before playback commences. For streaming, the video playback commences before the download of the entire video is completed; preferably, playback should commence as soon as possible after the request for the video. Once playback commences, a new video frame needs to be received, decoded, and displayed at the frame rate of the video, for example, 30 frames/s, to ensure uninterrupted playback. This continuous playback requirement introduces timing constraints for streaming video; however, the preencoded nature of the video allows networking protocols to prebuffer (prefetch) video frames well ahead of their playback time so as to ensure continuous playback during periods of network congestion when the delivery of encoded video frames over the network slows down.

Live video transmission has two main subcategories, namely, live interactive video transmission, for example, from a video conference (conversation) between two or more participants, and live noninteractive video transmission, for example, from the video coverage of a sporting event. For live interactive video, the one-way end-to-end delay, including the delays for video encoding, network transmission, and video decoding, should preferably be less than 150 ms to preserve the interactive conversational nature of the communication. For live noninteractive video, it is typically permissible to have some lag time between the capture of the live event and the playback of the video at the receivers to accommodate video encoding, network transport, and decoding. However, due to the live nature it is not possible to prefetch video frames as in the case of video streaming.

The H.264/AVC and H.265/HEVC standards offer a wide range of encoding options to accommodate the timing and other constraints (e.g., computational capabilities) of the different video transmission scenarios. The encoding options can be flexibly employed to suit the needs of the particular video transmission scenario. For instance, for live interactive video transmission, low-delay encoding options arrange the inter-frame dependencies to permit fast encoding of video frames from a live scene so as to avoid extensive delays due to waiting for the capture of future video frames [51]. Such low-delay encoding options slightly reduce the efficiency of inter-frame prediction and thus slightly reduce the RD efficiency of the encoding. On the other hand, transmission scenarios with relaxed timing constraints, such as live noninteractive video, as well as video download and streaming, can employ the full inter-frame prediction options with hierarchical B frames, for example, with the dyadic prediction structure in Figure 2(b). In summary, not all encoding tools and refinements of these coding standards need to be employed; rather only those encoding tools and refinements that are appropriate for a given video network transport scenario can be employed.

### 3.2. Video Traffic Characteristics at Encoder Output

**Figure 3:**
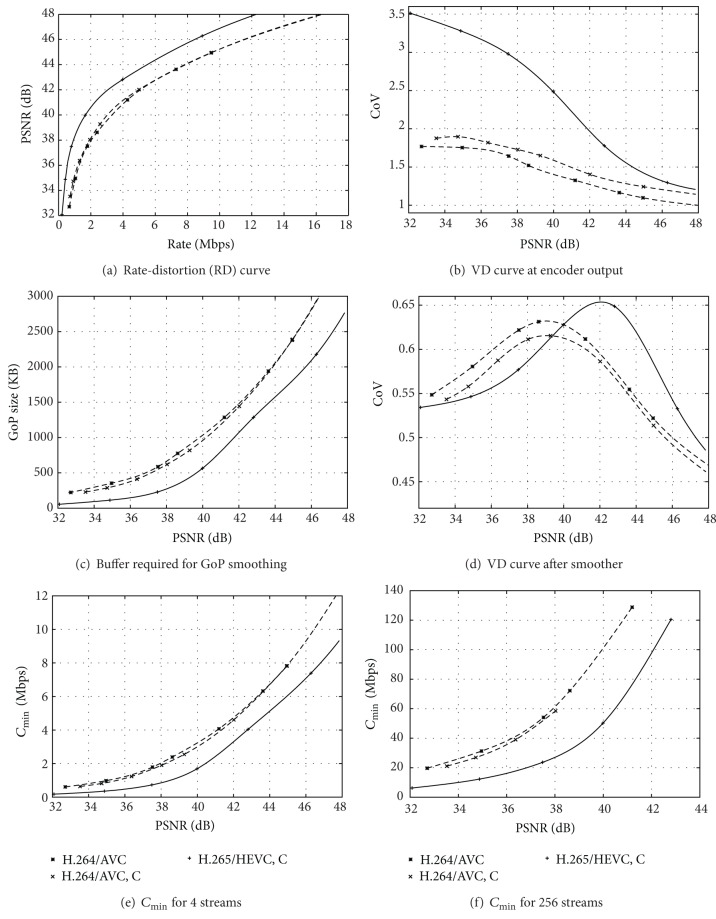
Traffic characteristics and link bandwidth requirements for H.264/AVC without and with cascading (C) quantization parameters (QPs) and H.265/HEVC with cascading QPs for *Sony* video.

**Figure 4:**
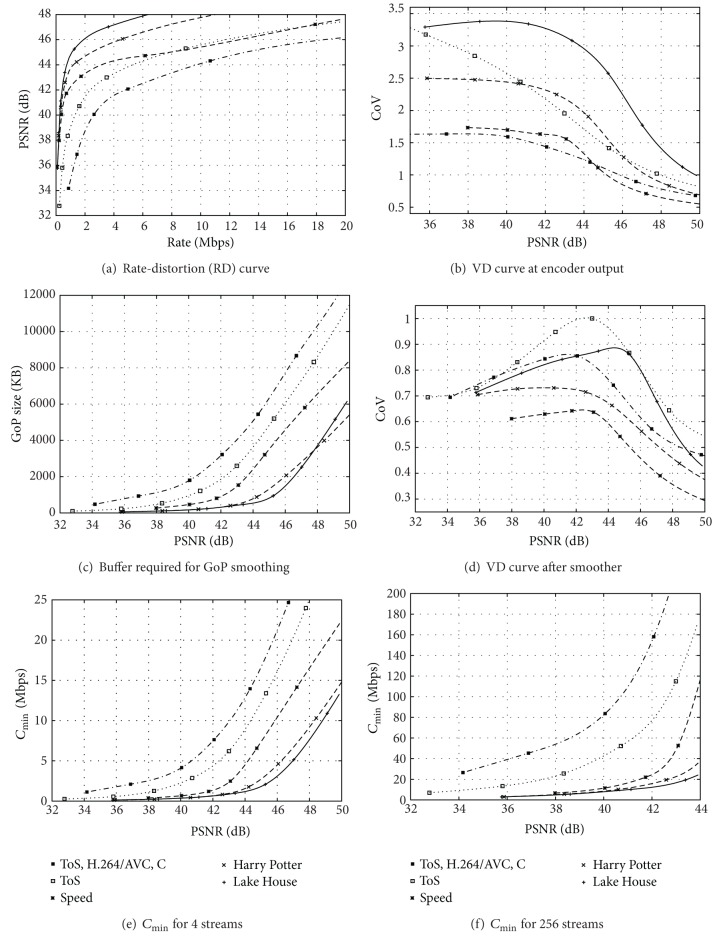
Traffic characteristics and link bandwidth requirements for H.265/HEVC with cascading QPs for a variety of videos, as well as comparison of H.265/HEVC (with cascaded QPs) with H.264/AVC (with cascaded QPs) for *Tears of Steel (ToS)* video.

In this section, we focus on video transmission scenarios with relaxed timing constraints. We present traffic characteristics of H.264/AVC and H.265/HEVC video streams for the 10-minute (17,682 frames) *Sony Digital Video Camera Recorder* demo sequence in Figure 3. The *Sony Digital Video Camera Recorder* demo sequence, which we refer to as *Sony* sequence in short, is a widely used video test sequence with a mix of scenes with high texture content and a wide range of motion activity levels. The *Sony* sequence has a frame rate of 30 frames/s, that is, a frame period of 1/30 s. We present H.264/AVC and H.265/HEVC traffic characteristics for the *Tears of Steel* video, which we abbreviate to *ToS* in Figure 4. The *ToS* video has 17,620 frames with a frame rate of 24 frames/s and is a combination of real movie scenes shot in natural environments overlaid with computer-generated graphics. The *ToS* movie depicts a futuristic science fiction battle between humans and robots. Moreover, we present in Figure 4 the H.265/HEVC video traffic characteristics for the first hour (86,400 frames at 24 frames/s) of each of the following movies: *Harry Potter*, *Lake House*, and *Speed*. *Harry Potter* depicts fiction content about a wizard apprentice with a variety of life-like special effects and changing dynamics and scene complexity. *Lake House* is a generally slow-paced romantic drama movie. *Speed* is a fast-paced action thriller with high content dynamics throughout. We consider all videos in the full HD 1920 × 1080 pixel format. Video traces and plots for these representative videos and other videos are available from http://trace.eas.asu.edu/.

In Figures 3(a) and 4(a), we plot the RD curves, that is, the video quality as a function of the mean bitrate, obtained with coding standard reference software implementations. For the single-layer encodings, we employ a GoP structure with 24 frames, specifically, one I frame and 3 P frames, as well as a dyadic prediction structure of with *β* = 7 B frames between successive I and P frames, analogous to Figure 2(b). We represent the video quality in terms of the peak signal to noise ratio (PSNR) between the luminance values in the sequence of original (uncompressed) video frames and the sequence of encoded (compressed) and subsequently decoded video frames. The PSNR is a rudimentary objective video quality metric; for an overview of video quality metrics, we refer to [52]. In Figures 3(b) and 4(b), we plot the rate variability-distortion (VD) curve defined as a plot of the coefficient of variation (CoV) of the encoded frame sizes (in Bytes) [17, 53, 54], that is, the standard deviation of the frame sizes normalized by the mean frame size, as a function of the PSNR video quality.

We observe from Figure 3(a) that H.264/AVC with QP cascading (C) slightly improves the RD efficiency, that is, increasing the PSNR video quality for a prescribed (specific) mean bitrate, compared to encoding without QP cascading, while increasing the traffic variability, as observed in Figure 3(b). The QP cascading leads to increasing compression for higher levels of the B frame hierarchy, which increases RD efficiency as these B frames in the higher layers are used as references for fewer other B frames. However, the interspersing of more intensely compressed frames in between other less compressed frames increases the variability of the encoded frame sizes. We note that video traffic variations both over short-time scales, as conducted here, as well as long-time scales, which reflect to a large degree the content variations of the encoded videos, have been studied for the past 20 years, mainly for the early MPEG codecs [55–57]. To the best of our knowledge, the effects of QP cascading in H.264/AVC on the traffic characteristics of long videos are for the first time examined in Figure 3.

Similarly, we observe from Figures 3(a) and 3(b) as well as Figures 4(a) and 4(b) increased RD efficiency and higher frame size variability with H.265/HEVC compared to H.264/AVC. Specifically, we observe from Figures 3(a) and 4(a) that H.265/HEVC gives approximately 2 dB higher average PSNR video quality compared to H.264/AVC for a wide range of encoding bitrates. The CoV values for H.264/AVC reach close to two for *Sony* in Figure 3(b) and slightly above 1.5 for *Tears of Steel (ToS)* in Figure 4(b), while H.265/HEVC gives CoV values reaching close to 3.5 for these two videos. Overall, the results in Figures 3(a) and 3(b) indicate that the H.265/HEVC standard allows for the transmission of higher quality video with lower mean bitrates compared to H.264/AVC. However, the network needs to accommodate higher fluctuations of the bitrates required to transport the encoded frame sequence. We examine in Sections 3.3 and 3.4 how elementary smoothing and multiplexing mechanisms translate the higher RD efficiency of H.265/HEVC into reduced link bandwidth requirements.

We observe for the different H.265/HEVC encoded videos in Figures 4(a) and 4(b) that the video content greatly affects the RD and VD characteristics at the encoder output. We observe that *Lake House* not only gives the highest RD efficiency but also the highest CoV values. On the other hand, *ToS* gives the lowest RD efficiency and *Speed* the next to lowest RD efficiency in Figure 4(a), while *Speed* gives the lowest CoV values in Figure 4(b). Generally, the RD and VD characteristics of a video encoding are influenced to a large degree by the motion and texture characteristics of the video content [58–61]. *Lake House* contains long stretches of relatively low-motion content with low to moderate texture complexity, allowing for highly RD-efficient compression. On the other hand, *Lake House* has a few high-motion scenes interspersed within the generally slow-moving scene content. This mixing of high and slow motion scenes results in relatively high traffic variability at the encoder output.

In contrast, *Speed* features quite consistently high-motion content in most scenes, while *ToS* has relatively high texture complexity due to the overlaying of natural scenes with computer-generated graphics in addition to high motion content in many fast-changing scenes. As a result, these two videos are relatively more difficult to compress and give lower RD efficiency, as observed in Figure 4(a). The consistently high motion content in *Speed* implies also relatively low variability (CoV) of the traffic bitrate at the encoder output, as observed in Figure 4(b). We also observe that *Harry Potter* is in the midrange of the RD and VD values in Figures 4(a) and 4(b). These midrange characteristics are due to the relatively balanced mix of low to high motion scenes and the moderate texture complexity in most scenes in *Harry Potter*.

### 3.3. Smoother

In order to ensure continuous video playback, a new video frame needs to be displayed every frame period. Network congestion may delay the delivery of encoded video frames to the receiver. Systems for video streaming and live noninteractive video transmission mitigate the effects of network congestion by buffering some video frames in the receiver before commencing video playback. Moreover, buffering helps in reducing the high variability of the frame sizes (i.e., the video bitrate) at the encoder output by smoothing out the frame size variations. A wide array of video smoothing techniques has been researched for video encoded with the early MPEG codecs [62–67] so that variable bitrate encoded video can be more easily transported over networks.

For examining the smoothing effect on H.264/AVC and H.265/HEVC encoded video, we consider elementary smoothing over the frames in each GoP. That is, the 24 frames in a GoP are aggregated and are transmitted at a constant bitrate corresponding to the mean size of a frame in the GoP divided by the frame period. In Figures 3(c) and 4(c), we plot the maximum GoP size (in kByte), which corresponds to the buffer required in the smoother in Figure 1 (a complementary buffer is required in the receiver for undoing the smoothing). We observe that H.265/HEVC has significantly lower buffer requirements than H.264/AVC. The higher frame size variations of H.265/HEVC compared to H.264/AVC as observed in Figures 3(b) and 4(b) do *not* result in higher buffer requirements. Rather, the lower mean bitrate of H.265/HEVC compared to H.264/AVC for a specific mean PSNR video quality, see Figures 3(a) and 4(a), dominates to result in lower buffer requirements for H.265/HEVC.

Similarly, we observe for the H.265/HEVC encodings in Figure 4(c) that *Lake House*, which has the lowest mean bitrates in Figure 4(a) and the highest CoV values at the encoder output in Figure 4(b), has the lowest buffer requirements. More generally, the buffer requirement curves in Figure 4(c) for the different videos have essentially the inverse order of the RD curves in Figure 4(a), irrespective of the ordering of the VD curves in Figure 4(b). That is, among the different H.265/HEVC encodings, the mean bitrate dominates over the traffic variability to mainly govern the buffer requirements.

Figures 3(d) and 4(d) show the VD curve of the smoothed video traffic, that is, the coefficient of variation of the smoothed frame sizes, as a function of the mean PSNR video quality. We observe that the smoothing results in very similar traffic variabilities for the considered encoding approaches. The CoV differences between H.265/HEVC and H.264/AVC at the encoder output were above one in Figure 3(b) and above two in Figure 4(b) and are now at the smoother output below 0.05 in Figure 3(d) and below 0.12 in Figure 4(d).

We observe for the different H.265/HEVC encodings in Figure 4 that the smoothing has reduced the CoV values from up to 3.5 at the encoder output (see Figure 4(b)), to less than one after the smoother (see Figure 4(d)). We also observe from Figures 4(b) and 4(d) that the relative order of the CoV curves is largely maintained by the smoothing, that is, the *Lake House* and *ToS* videos that had the highest CoV values at the encoder output (see Figure 4(b)), still have the highest CoV values after the smoother (see Figure 4(d)). On the other hand, *Speed* has the lowest CoV values and *Harry Potter* has intermediate CoV values across both Figures 4(b) and 4(d). An interpretation of these observations is that the smoothing mitigates the short-term traffic bitrate variabilities, but does not fundamentally alter the underlying long-term (GoP) time scale traffic variations.

### 3.4. Video Stream Multiplexing in Network

In many packet-switched networking scenarios, encoded and smoothed video streams are statistically multiplexed with each other and with other traffic streams at the network nodes. We study the statistical multiplexing effect for a single network link (modeling the bottleneck link in a larger network) with transmission bitrate *C* bit/s.

The link buffer can hold as much data as the link transmits in one frame period. In order to reveal the fundamental statistical multiplexing characteristics of the video encoding, we simulate the transmission of a fixed number of copies of the same encoded and smoothed video, each with its own random offset (starting frame index). We determine the minimum link bitrate *C*_min⁡_ that can support the fixed number of video streams while keeping the proportion of lost video information due to link buffer overflow below a minuscule 10^−5^. We assume that the error resilience mechanisms keep the impact of such minuscule losses on the video quality negligible.

Figures 3(e) and 3(f) as well as Figures 4(e) and 4(f) show plots of the minimum required link bandwidth *C*_min⁡_ for the multiplexing of 4 streams and 256 streams, respectively. We observe that H.265/HEVC has the lowest *C*_min⁡_ and that the reduction of *C*_min⁡_ becomes more pronounced when a larger number of streams are statistically multiplexed. Thus, we observe from these results that the gain in RD coding efficiency with the H.265/HEVC standard readily translates into reduced link bitrate requirements, or equivalently into an increased number or quality of transported video streams for a fixed link bitrate.

We furthermore observe for the different H.265/HEVC encodings in Figures 4(e) and 4(f) that the *C*_min⁡_ curves are essentially the inverse of the RD curves in Figure 4(a). That is, the mean bitrates largely govern the link bandwidth required for the transport of sets of multiplexed smoothed streams.

### 3.5. Timing Constraints due to Frame Dependencies

If the dyadic hierarchical B frame structure of H.264/AVC and H.265/HEVC is employed, it imposes additional constraints on the timing of the frame transmissions compared to the classical B frame prediction employed in the preceding MPEG-2 and MPEG-4 Part 2 standards. Generally, a given frame can only be encoded *after* all reference frames have been captured by a video camera and encoded and subsequently decoded and stored in the decoded frame buffer in Figure 1. For instance, in Figure 2(b), frame B_1_ can only be encoded after frames I_0_, P_8_, B_4_, and B_2_ have been encoded. In contrast, with classical B frame prediction illustrated in Figure 2(a), frame B_1_ can be immediately encoded after frames I_0_ and P_8_ have been encoded. Smoothing the encoded frames over groups of *a* frames, as well as the reordering of the frames from the encoding order to the original order in which the frames were captured, introduces additional delays. Live video requires all blocks depicted in Figure 1 and incurs all corresponding delays, which are analyzed in detail in [17] and give the total delay in Table 2. For prerecorded video, the server can directly send the smoothed video stream into the network, thus incurring only delays for network transmission, decoding, and frame reordering to give the original frame sequence. Overall, we note from Table 2 that the dependencies between B frames in the dyadic B frame hierarchy introduce an additional delay of [log⁡_2_(1 + *β*)] − 1 frame periods compared to the classical B frame prediction structure.

## 4. Overview of Scalable Video Encoding

### 4.1. Layered Video Encoding

MPEG-2 and MPEG-4 provide scalable video coding into a base layer giving a basic version of the video and one or several enhancement layers that improve the video quality. The quality layering can be done in the dimensions of temporal resolution (video frame frequency), spatial resolution (pixel count in horizontal and vertical dimensions), or SNR video quality. The layering in the SNR quality dimension employs coarse quantization (with high QP) for the base layer, and successively finer quantization (smaller QPs) for the enhancement layers that successively improve the SNR video quality. These layered scalability modes permit scaling of the encoded video stream at the granularity of complete enhancement layers; for example, a network node can drop an enhancement layer if there is congestion downstream. MPEG-4 has a form of sublayer SNR quality scalability referred to as fine grained scalability (FGS). With FGS there is one enhancement layer that can be scaled at the granularity of individual Bytes of video encoding information. With both MPEG-2 and MPEG-4, the flexibility of scaling the encoded video stream comes at the expense of a relatively high encoding overhead that significantly reduces the RD efficiency of the encoding and results in very limited adoption of these scalability modes in practice.

Similar to the preceding MPEG standards, the Scalable Video Coding (SVC) extension of H.264/AVC [6, 68] provides layered temporal, spatial, and SNR quality scalability, whereby the layered SNR quality scalability is referred to as Coarse Grain Scalability (CGS). While these H.264 SVC layered scalability modes have reduced encoding overhead compared to the preceding MPEG standards, the overhead is still relatively high, especially when more than two enhancement layers are needed.

### 4.2. H.264 SVC Medium Grain Scalability (MGS) Encoding

H.264 SVC has a novel Medium Grain Scalability (MGS) that splits a given SNR quality enhancement layer of a given video frame into up to 16 MGS layers that facilitate highly flexible and RD efficient stream adaption during network transport.

#### 4.2.1. MGS Encoding

As for all SNR quality scalable encodings, the base layer of an MGS encoding provides a coarse quantization with a relatively high QP; for example, B = 35 or 40. MGS encodings have typically one enhancement layer providing a fine quantization with a relatively small QP; for example, *E* = 25. When encoding this enhancement layer, the 16 coefficients resulting from the discrete cosine transform of a 4 × 4 block are split into MGS layers according to a weight vector (a similar splitting strategy is employed for larger blocks). For instance, for the weight vector **W** = [1,2, 2,3, 4,4], the 16 coefficients are split into six MGS layers as follows. The lowest frequency coefficient is assigned to the first MGS layer *m* = 1, the next two higher frequency coefficients are assigned to the second MGS layer *m* = 2, and so on, until the four highest frequency coefficients are assigned to the sixth MGS layer *m* = 6, the highest MGS layer in this example. For network transport, the base layer and each MGS layer of a given frame are encapsulated into a so-called network adaptation layer unit (NALU).

#### 4.2.2. Scaling MGS Streams in Network

When scaling down an MGS video stream at a network node, dropping MGS layers uniformly across the frame sequence results in low RD efficiency of the downscaled stream. This is due to the dependencies in the B frame hierarchy. Specifically, dropping an MGS layer from a B frame that other B frames depend on, for example, frame B_8_ in Figure 2(b), reduces not only the SNR quality of frame 8, but also of all dependent frames B_1_–B_7_ and B_9_–B_15_. It is therefore recommended to drop MGS layers first from the B frames without any dependent frames, that is, the odd-indexed B frames in the highest layer in Figure 2(b), then drop MGS layers from the B frames with one dependent B frame, that is, the B frames in the second highest layer in Figure 2(b), and so on [69].

Alternatively, MGS encodings can be conducted so that each NALU is assigned a priority ID between 0 indicating lowest priority and 63 indicating highest priority for RD efficiency. These priority IDs can be assigned by the video encoder based on RD optimization. For downscaling an MGS stream with priority IDs, a network node first drops MGS layers (NALUs) with priority ID 0, then priority ID 1, and so on.

## 5. Network Transport of H.264 SVC Video Streams

**Figure 5:**
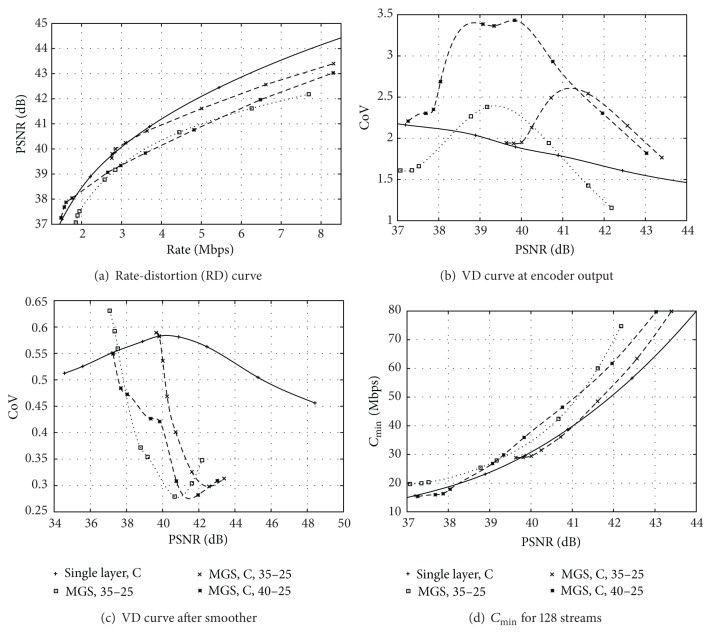
Illustration of traffic characteristics and link bandwidth requirements for *Sony* encoded with H.264 SVC with medium-grain scalability (MGS) for base layer QPs B = 35 and 40 and enhancement layer QP *E* = 25 with and without QP cascading (C), in comparison with H.264/AVC single-layer encoding with cascading QPs.

In Figure 5(a), we plot the RD curve of the *Sony* HD video for the priority ID stream scaling. We compare the RD curves of the MGS streams with cascaded QPs (C) with the RD curve from single-layer encoding, whereby all encodings have a GoP structure with one I frame and *β* = 15 B frames with the prediction structure illustrated in Figure 2(b). We observe that H.264 SVC MGS provides the flexibility of scaling the stream bitrate in the network with a low encoding overhead from the lower end to the midregion of the quality adaptation range between the base layer only and the base plus full enhancement layer. For instance, the RD curve of MGS, C encoding with B = 35, *E* = 25, in Figure 5(a) is very close to the RD curve of the single-layer encoding from its lower end near 39.7 dB through the lower third of the adaptation region up to about 41 dB. Near the lower end of the adaptation range, only the NALUs with the highest priority ID, that is, the highest ratio of contribution towards PSNR video quality relative to size (in Bytes) are streamed, resulting in high RD efficiency that can even slightly exceed the RD efficiency of the single-layer encoding. Towards the upper end of the adaptation range, all NALUs, even those with small PSNR contribution to size ratios are streamed, resulting in reduced RD efficiency. The difference in RD efficiency between the single-layer encodings and the MGS encoding at the upper end of the MGS adaptation range is mainly due to overhead of the MGS encoding.

Similar to the single-layer encoding, we observe from the comparison of MGS streams encoded without and with cascaded QPs in Figures 5(a) and 5(b) that the cascading increases both the RD efficiency and the traffic variability at the encoder output. We also observe that the cascaded-QPs MGS encoding with the larger adaptation range (B = 40, *E* = 25) gives somewhat lower RD efficiency and substantially higher traffic variability at the encoder output than the corresponding B = 35, *E* = 25 encoding. That is, the increased adaptation flexibility of the B = 40, *E* = 25 encoding comes at the expense of reduced RD efficiency and very high traffic variability reaching CoV values above 3.5 at the encoder output.

We observe from Figure 5(c) that smoothing over the frames in a GoP effectively reduces the traffic variability of the MGS streams. In the region where the MGS RD efficiency is close to the single-layer RD efficiency, for example, in the region from about 39.7 to 41 dB for the B = 35, *E* = 25 MGS, C encoding, the CoV values of the MGS encoding are close or slightly below the single-layer CoV values.

The *C*_min⁡_ plots in Figure 5(d) are essentially a mirror image of the RD curves in Figure 5(a), indicating that the excellent RD performance of H.264 MGS translates into commensurate low requirements for link bitrate. Overall, we observe that in the lower region of the adaptation region, H.264 MGS provides adaptation flexibility while requiring similarly low link bitrates as the corresponding single-layer encodings. Only toward the midregion and upper end of the adaptation range does the increased overhead of the scalable MGS encoding become significant and result in increased link bitrate requirements compared to single-layer encodings.

## 6. 3D Video Streams

### 6.1. Overview of 3D Video

3D video employs views from two slightly shifted perspectives, commonly referred to as the left view and the right view, of a given scene. Displaying these two slightly different views gives viewers the perception of depth, that is, a three-dimensional (3D) video experience. Since two views are involved, 3D video is also sometimes referred to as stereoscopic video. The concept of employing multiple views from different perspectives can be extended to more than two views and is generally referred to as multiview video.

### 6.2. 3D Video Encoding and Streaming

3D video streaming requires the transport of the two sequences of video frames resulting from the two slightly different viewing perspectives over the network to the viewer. Since the two views capture the same scene, their video frame content is highly correlated. That is, there is a high level of redundant information in the two views that can be removed through encoding (compression). The Multiview Video Coding (MVC) standard builds on the inter-coding techniques that are applied across a temporal sequence of frames in single-layer video coding to extract the redundancy between the two views of 3D video. More specifically, MVC typically first encodes the left view and then predictively encodes the right view with respect to the left view.

One approach to streaming the MVC encoded 3D video is to transmit the encoded left and right views as a frame sequence with twice the frame rate of the original video, that is, left view of first video frame (from first capture instant), right view of first video frame, left view of second video frame, right view of second video frame, and so on. Since the right view is encoded with respect to the left view, it is typically significantly smaller (in Bytes) and the sequence of alternating left and right views result in high traffic variability, as illustrated in the next section.

Another MVC streaming approach is to aggregate the left and right views from a given video frame (capture instant) into one *multiview frame* for transmission. The sequence of multiview frames has then the same frame rate as the original video.

An alternative encoding approach for 3D video is to sequence the left and right views to form a video stream with doubled frame frequency and feed this stream into a single-view video encoder, such as H.264 SVC or H.265/HEVC. This approach essentially translates the interview redundancies into redundancies among subsequent frames. Similar to MVC encoding, the two encoded views for a given capture instant can be transmitted sequentially or aggregated.

Yet another encoding alternative is to downsample (subsample) the left and right views to fit into one frame of the original video resolution. For instance, the 1920 × 1080 pixels of left and right views are horizontally subsampled to 960 × 1080 pixels so that they fit side-by-side into one 1920 × 1080 frame. This side by side approach permits the use of conventional 2D video coding and transmission systems but requires interpolation at the receiver to obtain the left and right views at the original 960 × 1080 pixel resolution.

### 6.3. RD and VD Characteristics of 3D Video Streams

**Figure 6:**
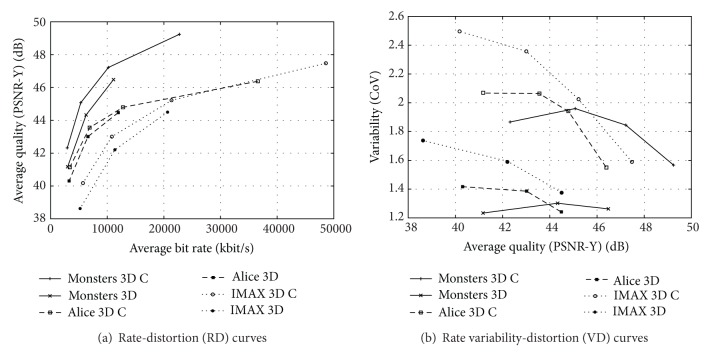
RD and VD characteristics of MVC encodings without and with cascaded QPs (C) of 35 minutes each of 3D videos *Alice in Wonderland* and *IMAX Space Station* with full HD 1920 × 1080 pixel resolution. The encoded left and right views are streamed sequentially (S) or are streamed aggregated (A) into multiview frames.

In Figure 6, we plot the RD and VD curves for two representative 3D videos encoded with MVC without QP cascading and with QP cascading. We employ the GoP structure with *β* = 7 B frames between successive I and P frames and 16 frames (i.e., one I frame, one P frame, and two sets of *β* = 7 B frames) per GoP. We observe from Figure 6(a) that (i) for a prescribed mean bitrate, the PSNR video quality is higher for the *Alice* video compared to the *IMAX* video and (ii) that the QP cascading improves the RD efficiency by up to about 0.5 dB for the *Alice* video and almost 1 dB for the *IMAX* video. These different RD efficiency levels and RD efficiency increases with QP cascading are due to the different content of the two videos. The *IMAX* video is richer in texture and motion and thus “more difficult” to compress and higher RD efficiency gains can be achieved with improved coding strategies.

We observe from Figure 6(b) that (i) the more varied *IMAX* video gives higher frame size variability and that (ii) QP cascading increases the frame size variability, mirroring the above observations for single-view (2D) video. We also observe from Figure 6(b) that the sequential (S) transmission of the encoded left and right views gives substantially higher traffic variability than the aggregated (A) transmission. Thus, the aggregated transmission with its less pronounced traffic fluctuations is typically preferable for network transport.

Recent studies [70] indicate that the frame sequential encoding results in somewhat lower RD efficiency and substantially lower traffic variability than MVC encoding. As a result, when statistically multiplexing a small number of unsmoothed 3D streams, MVC encoding and frame sequential encoding, both with the aggregated (A) transmission strategy, require about the same link bitrate. Only when statistically multiplexing a large number of streams, or employing buffering and smoothing, does MVC encoding reduce the required network bitrate compared to frame sequential encoding. The studies in [70] also indicate that the side-by-side 3D video approach gives relatively poor RD performance due to the involved subsampling and subsequent interpolation.

## 7. Conclusion

We have given an overview of modern video coding standards for multimedia networking. We have outlined the advances in the main video coding standards for nonscalable (single-layer) video and scalable video. For single-layer video, we gave an overview of H.264/AVC and H.265/HEVC. We compared their rate-distortion (RD) and rate variability-distortion (VD) characteristics before and after smoothing as well as link bitrate requirements. This comparison included the first study of the traffic variability (before and after smoothing) and statistical multiplexing characteristics of H.265/HEVC encoding for long videos as well as an original study of the effects of cascading of quantization parameters (QPs) for the different levels of the hierarchical dyadic B frame prediction structure of H.264/AVC. We found that the advances in the video coding standards have led to increased RD efficiency, that is, higher video quality for a prescribed mean video bitrate, but also substantially increased traffic variability at the encoder output (which is effectively mitigated through smoothing). We also found that elementary smoothing with moderately sized buffers and statistical multiplexing during network transport translate the RD improvements of H.265/HEVC into commensurate reductions of the required network link bitrate (for a given PSNR video quality).

For scalable video coding, we gave a brief overview of H.264 SVC Medium Grain Scalability (MGS) and the scaling of an encoded H.264 SVC MGS video bitstream in a network node. We compared the RD and VD characteristics (before and after smoothing) as well as the link bitrate requirements of the scaled H.264 MGS stream with corresponding single-layer encodings. We illustrated that H.264 SVC MGS streams can be flexibly scaled in the network in the lower region of the quality adaptation range while maintaining RD efficiency and link bitrate requirements very close to the unscalable single-layer encodings. For 3D video, we outlined the encoding and streaming of the two views and examined the RD and VD characteristics of the streams.

The H.264/AVC video coding standard formed the basis for the development of the highly efficient scalable video coding (SVC) extension as well as extensions for stereo and multiview video coding suitable for 3D video [8]. Similarly, H.265/HEVC currently forms the basis for ongoing developments of scalable video coding extensions and multiview video coding extensions [71–73]. There are many important research directions on communications and networking mechanisms for efficiently accommodating video streams encoded with modern encoding standards. The pronounced traffic variabilities of the modern video coding standards requires careful research on adaptive transmission with buffering/smoothing [74–80] as well as traffic modeling [81] and transport mechanisms. For instance, metro/access networks [82–85] that multiplex relatively few video streams may require specialized protocols for video transport [86–89].

## Figures and Tables

**Table 1:** Summary of main terminology and notations.

AVC	Advanced Video Coding
Avg. video bitrate (bit/s)	Average (mean) of frame sizes of frames in a video sequence divided by frame period
*β*	Number of bidirectional predicted (B) frames between successive I (or P) frames
CoV	Coefficient of variation, that is, mean value of a random quantity divided by its standard deviation
DCT	Discrete cosine transform
Frame size (Byte)	Number of Bytes of information to represent an encoded video frame
Frame period (s)	Display duration (in seconds) for a video frame, that is, inverse of frame rate (in frames/second)
HEVC	High Efficiency Video Coding
MVC	Multiview Video Coding
QP	Quantization parameter
RD curve	Rate-distortion curve, that is, plot of distortion (typically represented through PSNR video quality)
	as a function of average video bitrate
PSNR	Peak signal to noise ratio (dB)
PSNR video quality (dB)	Average (mean) of PSNR values of encoded frames in a video sequence
SVC	Scalable Video Coding
*τ*	Number of layers in hierarchical B frame structure; τ = log⁡_2_(β + 1) for dyadic hierarchy
VD curve	Rate variability-distortion curve, that is, plot of rate variability (typically represented by CoV of frame sizes)
	as a function of video distortion (typically represented by PSNR video quality)

**Table 2:** End-to-end delay in frame periods for video encodings with β, β ≥ 1, B frames between successive I/P frames and smoothing over *a* frames from [17]; encoding, network transport, and decoding are assumed to each requiring one frame period per frame.

	Live video	Prerecorded video
Classical B frame prediction	β + 2*a* + 2	*a* + 2
Hierarchical B frame prediction	β + 2*a* + 1 + log⁡_2_(β + 1)	*a* + 1 + log⁡_2_(β + 1)
